# Alterations in network robustness upon simultaneous temperature and pH perturbations

**DOI:** 10.1152/jn.00483.2023

**Published:** 2024-01-24

**Authors:** David Hampton, Sonal Kedia, Eve Marder

**Affiliations:** Biology Department and Volen Center, Brandeis University, Waltham, Massachusetts, United States

**Keywords:** CPG, Cancer borealis, oscillatory neurons, pyloric rhythm, stomatogastric ganglion

## Abstract

Nervous systems have evolved to function consistently in the face of the normal environmental fluctuations experienced by animals. The stomatogastric nervous system (STNS) of the crab, *Cancer borealis*, produces a motor output that has been studied for its remarkable robustness in response to single global perturbations. Changes in environments, however, are often complex and multifactorial. Therefore, we studied the robustness of the pyloric network in the stomatogastric ganglion (STG) in response to simultaneous perturbations of temperature and pH. We compared the effects of elevated temperatures on the pyloric rhythm at control, acid, or base pHs. In each pH recordings were made at 11°C, and then the temperature was increased until the rhythms became disorganized (“crashed”). Pyloric burst frequencies and phase relationships showed minor differences between pH groups until reaching close to the crash temperatures. However, the temperatures at which the rhythms were disrupted were lower in the two extreme pH conditions. This indicates that one environmental stress can make an animal less resilient to a second stressor.

**NEW & NOTEWORTHY** Resilience to environmental fluctuations is important for all animals. It is common that animals encounter multiple stressful events at the same time, the cumulative impacts of which are largely unknown. This study examines the effects of temperature and pH on the nervous system of crabs that live in the fluctuating environments of the Northern Atlantic Ocean. The ranges of tolerance to one perturbation, temperature, are reduced under the influence of a second, pH.

## Graphical abstract

**Figure:**
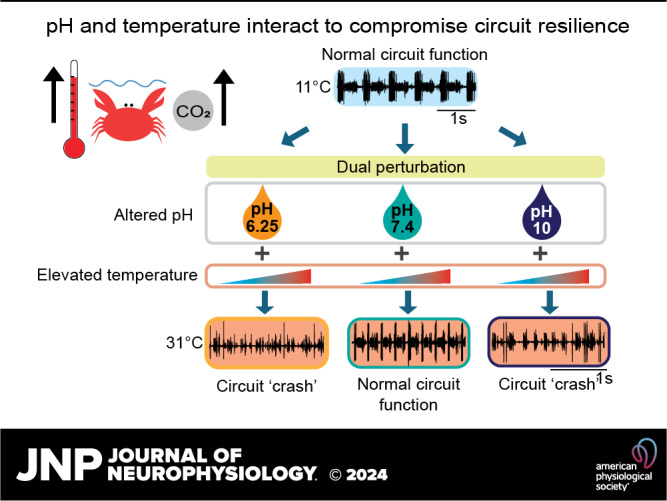

## INTRODUCTION

Animals often face large fluctuations in their external and internal environments. In mammalian species, homeostatic processes that maintain relatively constant internal environments are essential to life (1). For example, an increased internal temperature of 4°C can cause convulsions and death (2). Similarly, pH is tightly regulated (7.1–7.4) in most mammalian tissues (3), but microenvironments with pH as low as 5.8 are seen in injured or malignant tissues (4). Acidosis has been implicated in ischemia, seizures, and respiratory problems (5). Metabolic and respiratory disorders can lead to acidosis or alkalosis, which in humans can be fatal below a pH of 7.0 or above a pH of 7.8.

Most poikilotherms live in habitats that fluctuate in temperature. Many species show strong temperature preferences (6, 7), and travel in the environment to attempt to regulate their temperature. Many species also exhibit chemotaxis, which can include movement along a pH gradient to a preferred chemical environment (8).

The stomatogastric nervous system (STNS) of the crab, *Cancer borealis*, has been used to study the effects of both temperature and pH (9–16) on rhythmic motor patterns. *C. borealis* deals with large seasonal temperature swings and daily shifts of up to 10°C (17–19). Although deep ocean pH only naturally varies by a few tenths of a point (20), coastal and estuarine intertidal areas can reach a pH as low as 2–6 (21) due to agricultural run-off, navigation, and industrial and domestic effluents. Even in natural conditions, both ocean pH and crab hemolymph depend on bicarbonate buffers that are affected by dissolved CO_2_, which varies as a function of sea-water depth and temperature.

Relatively few studies have determined how exposure to one environmental perturbation influences the response to another perturbation (12). This study asks whether the robustness of the *C. borealis* pyloric rhythm is affected when temperature and pH are changed together, compared with the effects of either perturbation in isolation.

## METHODS

### Animals

Male Jonah crabs (*C. borealis*) weighing 400–700 g were bought from Commercial Lobster (Boston, MA). Animals were housed in tanks with Instant Ocean at 10–13°C on a 12-h light/dark cycle without food. Crabs were kept on ice for 30 min before dissection. Data from 29 crabs were used during the analysis.

### Saline Solutions

Control *C. borealis* physiological saline was as follows: 440 mM NaCl, 11 mM KCl, 13 mM CaCl_2_, 26 mM MgCl_2_, 11 mM Trizma base, and 5 mM maleic acid, pH 7.4–7.5 when measured at room temperature (RT) (20.5–23°C). Concentrated HCl and NaOH were added to achieve solutions of pH 6.25 (Acid) or 10.0 (Base) at RT. To determine the effect of temperature on the pH of each solution, the pH of a sample of each solution was measured twice at each temperature (over two temperature ramps/solution), and the measurements were averaged (Table 1). As temperature changed, the pH of each solution changed (Table 1), which also occurs in crustacean hemolymph (22). Therefore, each pH condition referred to represents the pH range across temperature. Solution pH was measured using a calibrated pH/ion meter (Mettler Toledo S220).

**Table 1.** pH of solutions at each temperature

*T*, °C	Control	Acid	Base
11	7.81	6.26	10.31
15	7.68	6.26	10.18
19	7.57	6.26	10.06
23	7.48	6.26	9.94
25	7.41	6.23	9.87
27	7.36	6.22	9.81
29	7.29	6.22	9.74
31	7.24	6.21	9.67
33	7.19	6.20	9.61
35	7.14	6.20	9.53

### Experimental Protocol

The STNS was dissected and pinned in a Sylgard (Dow Corning) coated Petri dish. The sheath surrounding the stomatogastric ganglion (STG) was partially opened to ensure that STG neurons were exposed to the solution applied. A Peltier device (Warner Instruments) was used to control the bath temperature. The saline inflow was placed close to the STG, with a temperature probe between the inflow and the STG.

Stainless steel pin electrodes recorded extracellular action potentials from Vaseline wells placed around nerves using a differential amplifier (A-M Systems Model 1700). Recordings were made from lateral ventricular nerves (*lvn*), pyloric dilator nerves (*pdn*), and pyloric nerves (*pyn*). Gravity-fed saline (∼10 mL/min) was superfused over the preparation. Baseline recordings were taken at 11°C. Preparations were maintained in altered pH saline for 25 min before the temperature ramp. Each preparation was used for one temperature ramp at a specified pH. For each condition, 2-min recordings were taken at temperature steps of 11°C, 15°C, 19°C, 23°C, 25°C, 27°C, 29°C, 31°C, 33°C, and 35°C followed by a 25-min recovery at 11°C. To prevent damage by high temperatures, ramps were terminated and brought back to 11°C after the pyloric dilator (PD) or lateral pyloric (LP) neurons stopped firing for more than 10 s, or regular bursting activity was lost for more than 10 s.

### Activity States

The activity was scored in cycles from the start of one PD burst to the start of the next. LP was determined on a *lvn*. PD activity was determined on a *pdn*.

MATLAB scripts were written to categorize activity. The spike time of the first PD spike in a burst was recorded as the start of the cycle. The end of a cycle was the spike time of the first spike of the following burst. All non-normal triphasic cycles were verified by eye. For phase relationships time was normalized to the duration from the first PD spike of a triphasic cycle to the first PD spike of the next cycle.

### Data Acquisition and Analysis

Data were acquired using a data acquisition board (Molecular Devices Digidata 1440A) and Clampex 10.5 software (Molecular Devices). Data were analyzed using Clampfit 10.5, Spike2 v 7.00 (Cambridge Electronic Design), and/or MATLAB 2018 A (MathWorks). Figures were prepared in Adobe Illustrator CC 2023. Spike2 scripts were used to extract burst information for LP, PD, and pyloric (PY) from 2-min files. MATLAB scripts were used to determine and plot PD burst frequency. SPSS was used to perform statistical analyses.

## RESULTS

### Temperature Robustness in Altered pH

**Figure 1.**
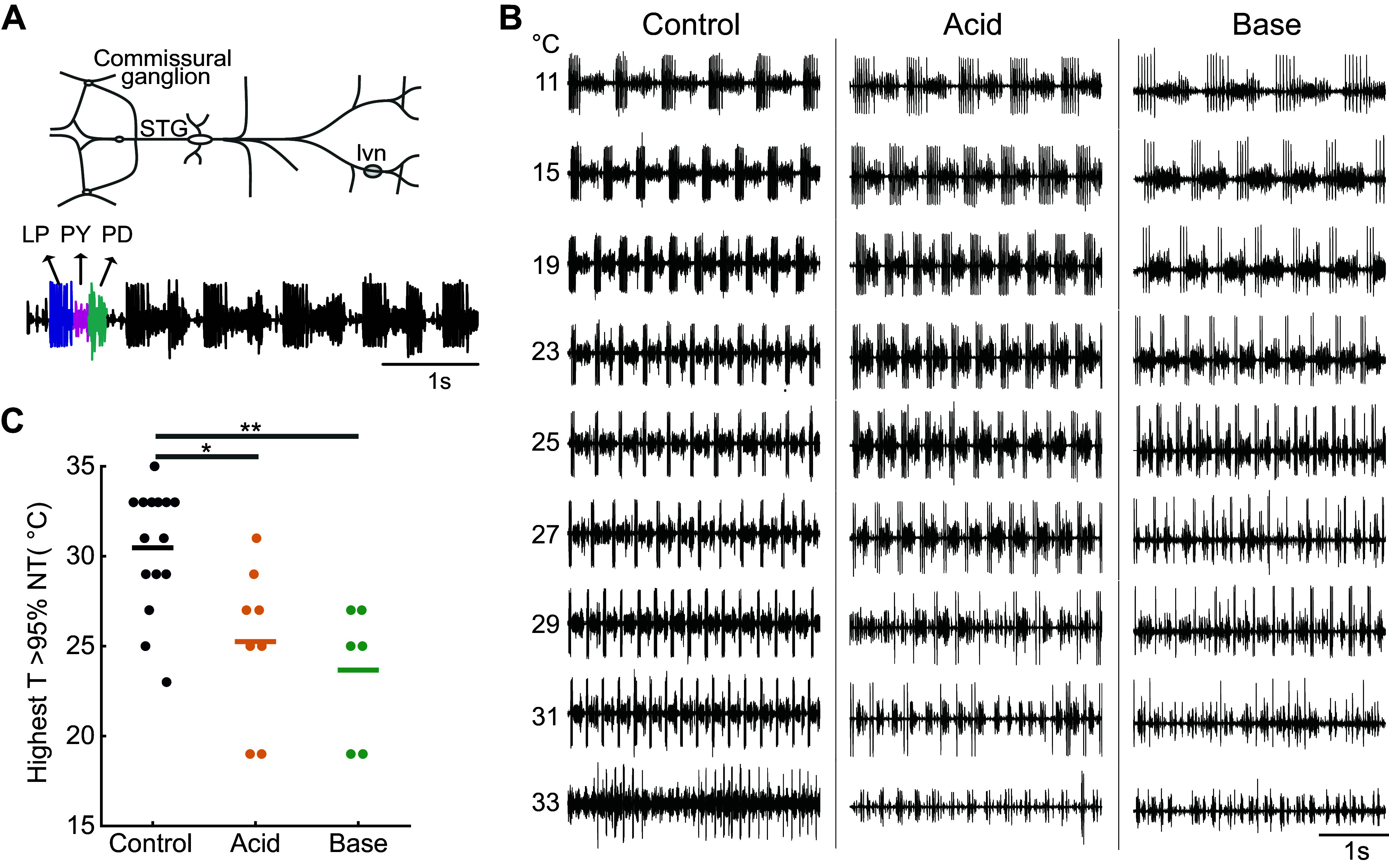
The effects of temperature and pH. *A*: diagram of the dissected stomatogastric nervous system (STNS). *Bottom*: lateral ventricular nerves (*lvn*) recording of normal triphasic activity with the spikes from individual pyloric circuit neurons identified. *B*: example traces at different pH conditions. Each column was taken from a different preparation in the specified pH condition. *C*: distributions of highest temperatures at which preparations display >95% normal triphasic activity at different pHs (control, acid, and base). Each dot represents a single preparation. Horizontal lines indicate population means (*P < 0.05, **P < 0.01). LP, lateral pyloric; NT, normal triphasic; PD, pyloric dilator; PY, pyloric; STG, stomatogastric ganglion.

Our goal was to examine the robustness of the *C. borealis* pyloric rhythm to simultaneous shifts in pH and temperature. Pyloric circuit activity was recorded extracellularly from the *lvn* (Fig. 1*A*). A control, normal triphasic pyloric rhythm (NTs) consists of a repeated sequence of PD, LP, and PY (Fig. 1*A*). This rhythm has a frequency of ∼0.5–2 Hz at 11°C (11, 12) with a *Q*_10_ of ∼2 (15).

Temperature ramps from 11°C to ∼33°C were performed at three pHs—Control (pH 7.5), Acid (pH 6.25), and Base (pH 10).

Normal triphasic activity breaks down in a number of ways, seen as obvious changes in the spiking activity of the pyloric neurons such as in their relative timing or consistency within and across cycles. Example traces in Fig. 1*B* demonstrate normal triphasic activity in control conditions from 11°C to 31°C and disrupted triphasic activity at 29°C in acid conditions (LP silent for several cycles), and at 27°C in Base (one or more pyloric units with 0–1 spikes/burst in several cycles).

We compared the highest temperature at which preparations exhibited normal triphasic activity for greater than 95% of the time (>NT95) across groups (Fig. 1*C*). A significant difference was observed between groups using a one-way ANOVA [*F*(2,26) = [9.28], *P* = 0.0009], and post hoc Tukey honestly significant difference (HSD) test revealed significant differences between the control and acid groups (*P* = 0.01), and the control and base groups (*P* < 0.01). Thus, while at lower temperatures (<23°C), triphasic activity expression appears unaltered, the range of temperatures at which preparations are still able to have significant triphasic activity is reduced in altered pH conditions.

### Analysis of Activity Disruption

**Figure 2.**
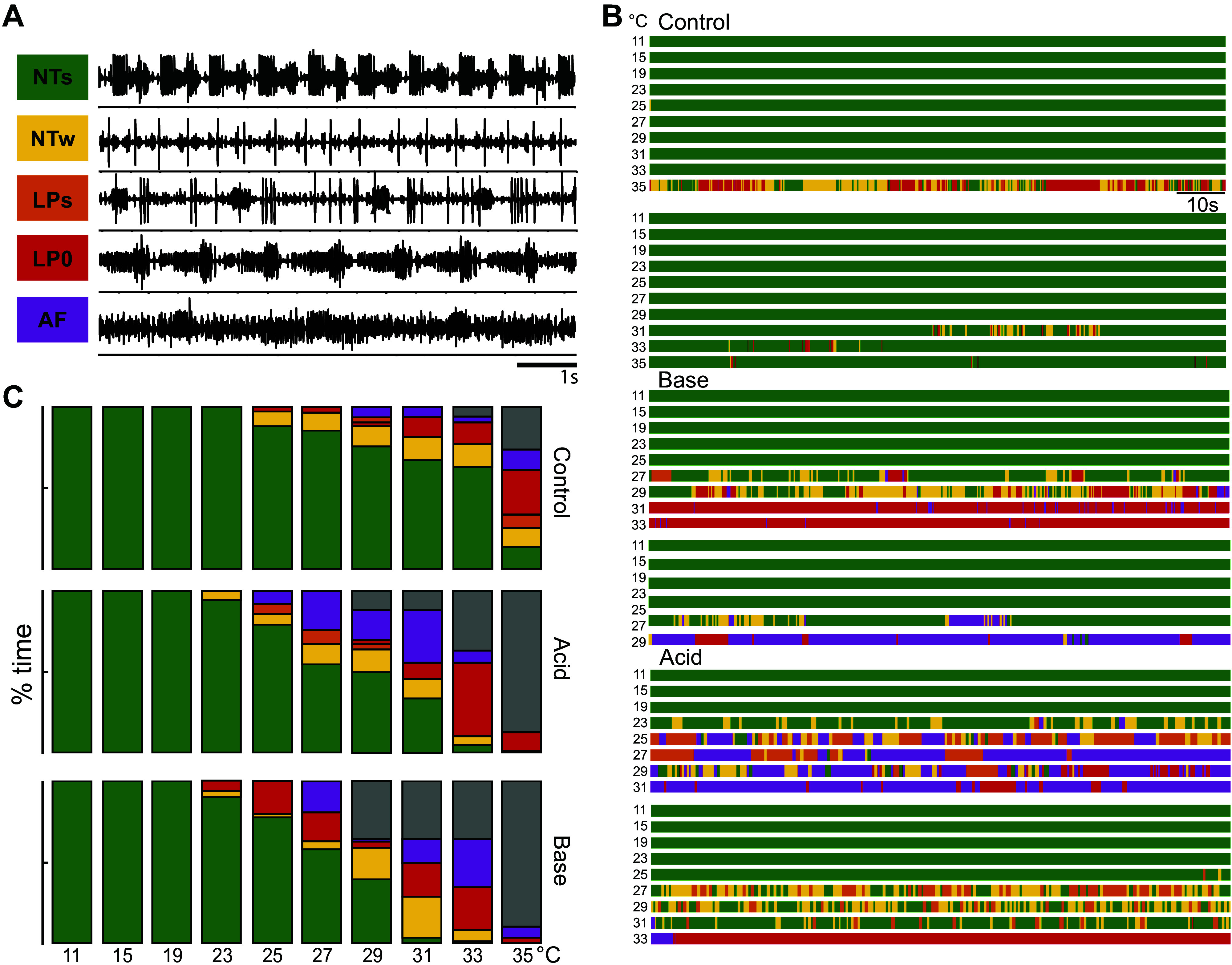
Network state analysis in altered pH and temperature. *A*: categories of normal triphasic strong (NTs), normal triphasic weak (NTw), lateral pyloric (LP) spread (LPs), LP zero (LP0), and altered firing (AF) are represented with 8s lateral ventricular nerves (*lvn*) traces. The colored box designates the activity type. *B*: activity states of six experiments, two per pH condition across temperature steps. *C*: fraction of time spent in each activity state for a given temperature, averaged across all experiments in a given pH condition. Gray bars = not recorded (NR). Control, *n* = 15; Acid, *n* = 8; Base, *n* = 6.

Previous work attempted to capture the animal-to-animal variability in how the pyloric rhythm lost normal activity when perturbed by changes in temperature or pH (9, 12, 13, 16, 23). Here we used a scoring system to characterize non-triphasic activity types (Fig. 2*A*).

Scoring was done on a cycle-by-cycle basis to get an accurate measure of the percent of time spent in each activity state over a 2-minute recording. The pyloric activity was considered “Normal Triphasic Strong” (NTs) if it contained bursts with at least two spikes from PD, LP, and PY. Activity that is mostly rhythmic but has one or more units with 0–1 spikes/burst was scored as “Normal Triphasic Weak” (NTw). Additional LP bursts or spikes in a cycle with ordinary PY and PD activity were scored as “LP Spread” (LPs). No LP spikes in a cycle with ordinary PY and PD activity were scored as “No LP” (LP0). Any activity that does not fit one of the above categories was scored as “Altered Firing” (AF).

Example analyses using this system are plotted for two animals in each pH group in Fig. 2*B*. Activity states for a given temperature were variable across experiments, even within a group. In Control, one of the preparations became disrupted at 35°C, and the second preparation at 31°C (Fig. 2*B*). In contrast, both Acid preparations became disrupted at considerably lower temperatures, 23°C and 27°C, as did the ones in Base at 27°C and 25°C (Fig. 2*B*). Temperature ramps were terminated after temperature steps during which LP, PD, or both stopped firing for multiple cycles and the category “Not Recorded” (NR) was used for remaining higher temperatures (Control: 4/15; Acid: 7/8, Base: 6/7 at 35°C).

To compare activity across experiments, the fraction of time spent in each activity state for each temperature step was averaged across all experiments within a group (Fig. 2*C*). No preparations exhibited less than 95% Normal Triphasic Strong (<95NTs) activity at temperatures below 25°C in the control group (0/15). In contrast, <95NTs activity was observed at 23°C in some Acid (2/8) and Base (2/6) preparations (Fig. 2*C*). Altered LPs states were more prominent in acid conditions at 23 and 25°C [Acid (3/8), Base (0/6), Control (0/15) preparations] (Fig. 2*C*). Therefore, there may be differences in the ways preparations “crash” in different pH conditions.

### Burst Analysis across Experiments

Since normal triphasic activity is seen under all pH conditions in the temperature range from 11 to 25°C, we examined the finer nuances of burst characteristics within this range as indicators of network interactions.

**Figure 3.**
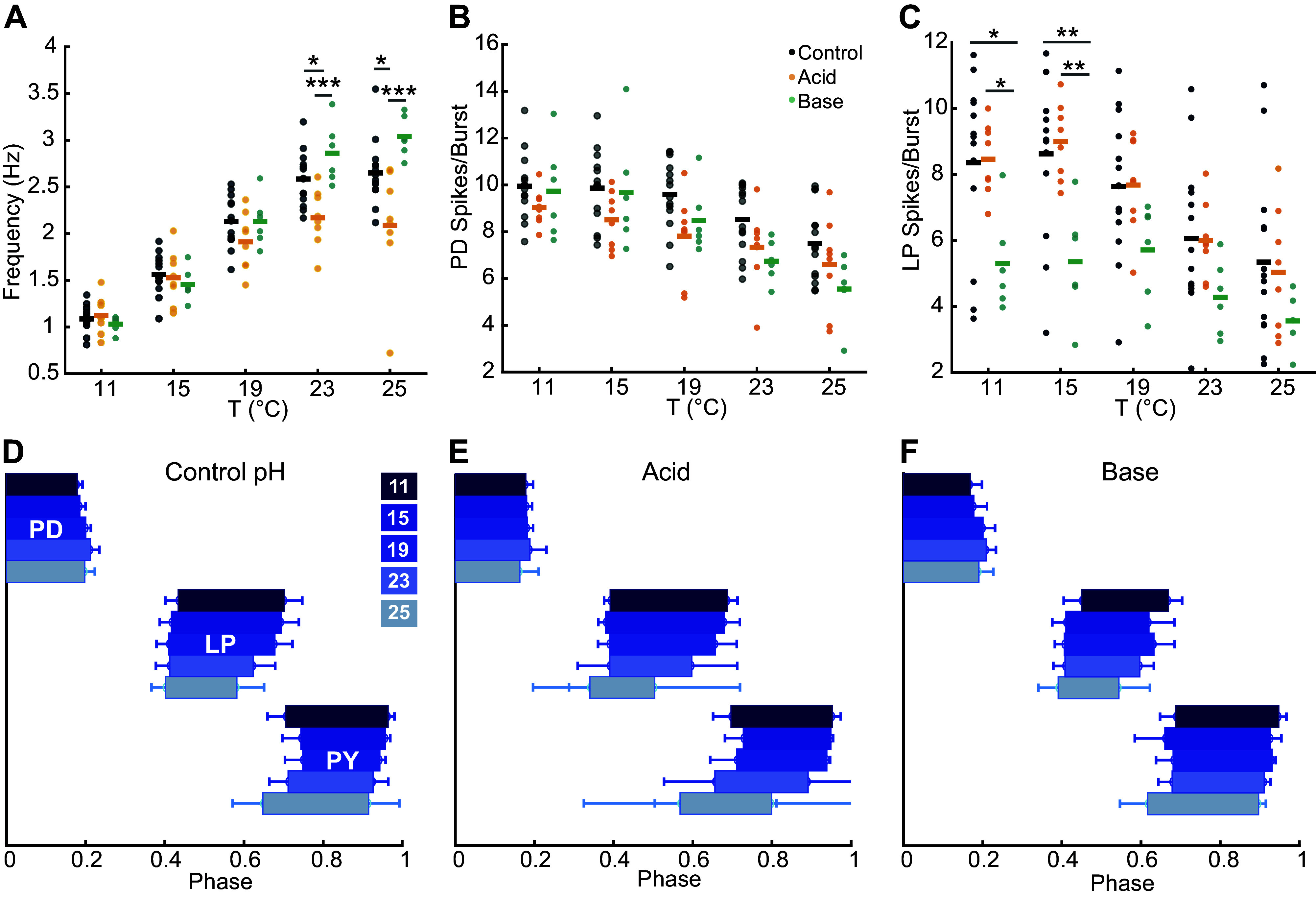
Similar bursting attributes between groups before activity degrades. *A*–*C*: attributes of burst analysis from 11°C to 25°C for each pH condition. Each dot represents data from an individual preparation with population means indicated by a solid line. *A*: burst frequencies at each pH and temperature. *B*: pyloric dilator (PD) spikes/bursts across temperatures were the same in different pHs. *C*: lateral pyloric (LP) spikes/bursts were lower in basic conditions at lower temperatures. (*B* and *C*: *P < 0.05, **P < 0.01, ***P < 0.001). *D*–*F*: the mean phase relationship between the starts and ends for PD, LP, and pyloric (PY) for each temperature in each pH condition. Bars represent the standard error across population means. No significant differences in any parameter across conditions.

We measured the mean burst frequency calculated from PD bursts for each preparation in each condition (Fig. 3*A*). Two-way repeated-measures mixed ANOVA found a significant effect of group and temperature [*F*(2,25) = 4.571, *P* = 0.02; *F*(1.71,42.92)= 8.85, *P* < 0.001 respectively]. Post hoc pairwise comparisons showed significant differences between control and acid and acid and base at 23°C (Control-Acid: *P* = 0.014, Acid-Base: *P* < 0.001) and at 25°C (Control-Acid: *P* = 0.018, Acid-Base: *P* < 0.001). This argues that at “permissive temperatures” the effects of pH are modest or nonexistent but become more apparent at extreme temperatures. No significant differences were observed across pH groups between 11 and 25°C in the number of PD spikes/burst (Fig. 3*B*). LP spikes per burst were compared using a mixed two-way repeated-measures ANOVA. There was a significant effect of pH group [*F*(2,25) = 4.825, *P* = 0.017] and temperature [*F*(4,100) = 41.351, *P* < 0.001]. Pair-wise comparisons with Bonferroni’s correction showed that basic conditions caused a significant decrease in LP spike numbers when compared with both control and acidic pHs at 11°C (Control-Base: *P* = 0.016; Base-Acid: *P* = 0.025) and at 15°C (Control-Base: *P* = 0.016; Base-Acid: *P* = 0.007) (Fig. 3*C*).

The relative timing of each neuron’s activity, or phase is approximately constant across frequencies for rhythms faster than ∼0.7 Hz (24–26), and is temperature invariant (14, 15). Despite differences in the number of spikes/bursts, phase relationships are conserved equally across pH groups (Fig. 3, *D–F*). The lack of difference in phase relationships across pH groups indicates that dual perturbation does not strongly impact the pyloric rhythm at temperatures below those at which activity begins to degrade.

## DISCUSSION

Organisms routinely face a number of environmental perturbations in their natural habitats, some of which occur simultaneously. This raises the issue of whether exposure to one perturbation compromises the animals’ ability to respond to additional perturbations. In this study, we examined the effects of temperature under three pH conditions and found that altered pHs degrade the crab’s ability to maintain reliable circuit output when additionally challenged with elevated temperatures while having no impact at control temperatures. This is potentially significant, as changes in temperature are often accompanied by changes in pH in biological systems, and it suggests that any resilience may be compromised more excessively by environmental stressors than can be easily predicted from the response to a single stressor.

Neuronal networks achieve robustness to environmental perturbation through both intrinsic and extrinsic mechanisms. In the pyloric network, the A-type current (*I*_A_), h-type current (*I*_h_), and synaptic currents (*I*_syn_) are important for phase constancy (15) and their interaction contributes to phase compensation over a wide range of temperatures, despite having different Q10’s (15). In other biological systems, changing pH has little effect on *I*_A_ and *I*_h_ in the range of our experiments (27, 28), but has a strong effect on excitatory glutamatergic synapses (29–31).

Other currents responsible for burst initiation or termination include Ca^2+^ current (*I*_Ca_) and Ca^2+^-dependent K^+^ current (*I*_KCa_). Both *I*_Ca_ (32, 33) and *I*_KCa_ (34, 35) are attenuated by acidic pH shifts. *I*_KCa_ is highly sensitive to changes in pH, with a p*K*_a_ close to that of physiological saline, whereas *I*_Ca_ has a p*K*_a_ closer to 5.2. This could explain the decrease in the number of LP spikes/bursts we saw across temperatures in the basic condition. Interestingly, recordings from populations of neurons in the mammalian brain exhibit the opposite trend, with increased activity in alkaline pH, and decreased activity in acidic pH (36). It is difficult to predict from the way activity is disrupted which parameters are compromised by extreme pH in the pyloric network because it is a highly degenerate system (37–39); that is, there is no direct relationship between parameter expression levels and the contribution of a particular parameter to specific portions of the activity waveform. In addition, the relative contribution of a parameter likely changes during a global perturbation, such as temperature (40, 41).

pH and temperature each affect protein conformation. pH affects hydrogen bonds, salt bridges, and electrostatic forces through charge interactions. Raising the temperature, or thermal energy, decreases the additional energy necessary for a protein to change its tertiary structure. These effects are additive. This may be why preparations in the acidic group recovered less often from temperatures above 27°C (data not shown). In less extreme conditions, dual perturbations could cause reversible protein conformation changes that lead to current attenuation whereas a single perturbation may not. Protein chaperones, such as heat shock proteins, mitigate some of the effects of environmental insult, including temperature and pH (42–44), by stabilizing the tertiary structure of proteins. Dual perturbations may push proteins to a point of deformation that is no longer viable for chaperone support.

Further work is needed to elucidate the mechanisms for how dual perturbations more strongly affect neuronal network activity than single perturbations. This study motivates further analyses, as it reveals that the combined effect of temperature and pH is greater than either alone. In the wild, intertidal crabs must experience multiple perturbations, as salinity and water oxygen tension also fluctuate, in addition to temperature and pH (45). Consequently, the animal may need to deal not just with two perturbations, but with multiple perturbations, which can occur together in a coordinated fashion. Future work will be needed to understand how these multiple environmental perturbations interact to influence the animal’s resilience to environmental challenges.

## GRANTS

This work was supported by National Institute of Neurological Disorders and Stroke Grant R35 NS097343.

## DISCLOSURES

No conflicts of interest, financial or otherwise, are declared by the authors.

## AUTHOR CONTRIBUTIONS

D.H. and E.M. conceived and designed research; D.H. performed experiments; D.H. and S.K. analyzed data; D.H. and S.K. interpreted results of experiments; D.H. and S.K. prepared figures; D.H. drafted manuscript; S.K. and E.M. edited and revised manuscript; S.K. and E.M. approved final version of manuscript.
